# Targeting antisense mitochondrial noncoding RNAs induces bladder cancer cell death and inhibition of tumor growth through reduction of survival and invasion factors

**DOI:** 10.7150/jca.38880

**Published:** 2020-01-17

**Authors:** Vincenzo Borgna, Lorena Lobos-González, Francisca Guevara, Eduardo Landerer, Maximiliano Bendek, Rodolfo Ávila, Verónica Silva, Claudio Villota, Mariela Araya, Alexis Rivas, Constanza López, Teresa Socias, Jorge Castillo, Luis Alarcón, Luis O. Burzio, Verónica A. Burzio, Jaime Villegas

**Affiliations:** 1 Fundación Ciencia & Vida,; 2 Facultad de Medicina, Universidad De Santiago,; 3 Servicio de Urología, Hospital Barros Luco-Trudeau,; 4 Centro de Medicina Regenerativa, Facultad de Medicina, Clínica Alemana, Universidad del Desarrollo,; 5 Andes Biotechnologies SpA,; 6 Facultad de Medicina, Universidad San Sebastián,; 7 Escuela de Nutrición y Dietética, Facultad de Salud, Universidad Bernardo O'Higgins,; 8 Facultad de Ciencias de la Vida, Universidad Andrés Bello,; 9 Servicio de Anatomía Patológica, Hospital Barros Luco-Trudeau. Santiago, Chile.

**Keywords:** bladder cancer, antisense therapy, noncoding RNA, apoptosis

## Abstract

Knockdown of the antisense noncoding mitochondrial RNAs (ASncmtRNAs) induces apoptotic death of several human tumor cell lines, but not normal cells, supporting a selective therapy against different types of cancer. In this work, we evaluated the effects of knockdown of ASncmtRNAs on bladder cancer (BCa). We transfected the BCa cell lines UMUC-3, RT4 and T24 with the specific antisense oligonucleotide Andes-1537S, targeted to the human ASncmtRNAs. Knockdown induced a strong inhibition of cell proliferation and increase in cell death in all three cell lines. As observed in UMUC-3 cells, the treatment triggered apoptosis, evidenced by loss of mitochondrial membrane potential and Annexin V staining, along with activation of procaspase-3 and downregulation of the anti-apoptotic factors survivin and Bcl-xL. Treatment also inhibited cell invasion and spheroid formation together with inhibition of N-cadherin and MMP 11. *In vivo* treatment of subcutaneous xenograft UMUC-3 tumors in NOD/SCID mice with Andes-1537S induced inhibition of tumor growth as compared to saline control. Similarly, treatment of a high-grade bladder cancer PDX with Andes-1537S resulted in a strong inhibition of tumor growth. Our results suggest that ASncmtRNAs could be potent targets for bladder cancer as adjuvant therapy.

## Introduction

Bladder cancer (BCa) is the most common malignant tumor of the urinary tract, corresponding to the seventh most common cancer worldwide, with over 549,000 new cases every year and accounting for over 199,000 deaths^1^. The most common type of BCa (70 - 80%) corresponds to urothelial carcinoma, a non-invasive papillary tumor that progress occasionally. About 20 - 30% of these cases develop into aggressive muscle-invasive carcinoma^2^, and approximately half of the patients will progress to metastatic disease with a 5-year survival rate of only 5%^3^. Cisplatin chemotherapy is recommended after radical nephroureterectomy^5^, albeit displaying high toxicity and low efficacy^4^-^6^. To improve survival rates, there is an urgent need to develop novel therapeutic strategies for BCa^7^.

We reported that the antisense non-coding mitochondrial RNAs (ASncmtRNAs) are promising targets to inhibit tumor growth in pre-clinical models. ASncmtRNA knockdown (ASK for short) with antisense oligonucleotides (ASO) targeted to the ASncmtRNAs, induces strong inhibition in tumor growth in syngeneic murine models of the aggressive murine melanoma cell line B16F10^8,9^ and the renal cell carcinoma line RenCa^10^. In addition, ASK in several human cell lines *in vitro*, using ASO Andes-1537, targeted to the human ASncmtRNAs, induces apoptotic cell death together with downregulation of survivin^11^, an anti-apoptotic factor that is overexpressed in tumor cells^12^,^13^. Interestingly, ASK does not affect normal cells, including human keratinocytes (HFK), endothelial cells (HUVEC), normal melanocytes (HnEM) and normal epithelial renal cells (HREC)^12^.

The ASncmtRNAs belong to a family of mitochondrial long non-coding RNAs containing stem-loop structures that also include a sense member (SncmtRNA)^14^-^16^. Interestingly; these transcripts exit the mitochondria to the cytosol and nucleus in normal and tumor cells suggesting a functional role outside the organelle^17^. SncmtRNA and ASncmtRNAs are differentially expressed according to proliferative status^15^; normal proliferative cells, but not resting cells, express the SncmtRNA and the ASncmtRNAs. Remarkably however, tumor cell lines and tumor cells in human biopsies of different origins downregulate the ASncmtRNAs^15^. Based on these results, we proposed that the ASncmtRNAs are tumor suppressors playing an important step in carcinogenesis and represent a new general hallmark of cancer, the last is due that ASncmtRNAs are down regulated, independent of tumor origin, therefore represent a universal signature such as, sustaining proliferative signaling, evading growth suppressors, resisting cell death, enabling replicative immortality, inducing angiogenesis, and activating invasion and metastasis^15^,^18^.

In the present work, we studied the effects of ASK on the human bladder cancer cell lines UMUC-3, RT4 and T24 and found that *in vitro* treatment induces a drastic inhibition of proliferation and apoptotic cell death. Moreover, ASK negatively affects the invasive capacity and spheroid formation of UMUC-3 cells, mediated by downregulation of N-cadherin and MMP11^19^,^20^. *In vivo* treatment with Andes-1537 of a UMUC-3 xenograft induced a strong inhibition of tumor growth, compared to controls. Similar results were obtained using a patient derived-xenograft (PDX) from a high-grade BCa patient. Altogether, these results indicate a potential use of the ASncmtRNAs as novel and potent adjuvant therapeutic targets for BCa.

## Materials and Methods

### Animal studies

Animal studies were conducted in accordance with the guidelines of Comisión Nacional de Investigación Científica y Tecnológica (CONICYT), Chile and approved by the Ethical Committee of Fundación Ciencia & Vida. Immuno-compromised NOD/SCID mice were obtained from Jackson laboratories (Bar Harbor, ME, USA) and maintained in the animal facility of the Fundacion Ciencia & Vida under specific pathogen-free conditions (Tecniplast, Buguggiate, Italy) in a temperature-controlled room with a 12/12 h light/dark schedule with sterile food and water *ad libitum.* Xenograft studies of BCa using the UMUC-3 cell line was carried out essentially as described before^8^,^10^. Briefly, 5 x 10^5^ UMUC-3 cells in 100 μl sterile saline were injected subcutaneously (sc) into the left flanks of 10 NOD/SCID mice and tumor growth was monitored every three days with an electronic caliper and tumor volume was estimated with the formula: tumor volume (mm^3^) = Length x Width^2^ x 0.5236. When tumors reached a volume of about 100 mm^3^, mice were randomized into two groups of 5 mice each. For treatment, mice were injected intraperitoneally (ip) every other day with 200 µl saline, either alone or containing 100 µg Andes-1537.

### Establishment of a BCa patient-derived xenograft (PDX) model

A tumor specimen was obtained from a patient diagnosed with BCa and subjected to radical bladder resection at the Urology Department of Hospital Barros Luco-Trudeau, Santiago, Chile. The protocol for this study was reviewed and approved by the Medical Ethics Board of the Hospital, together with the written informed consent of the patient and was performed in accordance with the principles of the Declaration of Helsinki. The pathology report indicated that the sample corresponded to stage T2N0M0. The tumor was preserved in sterile DMEM + antibiotics at 4°C and processed in the laboratory within 3 h. The tumor specimen was sliced into fragments of about 3 mm^3^ and 4 fragments were implanted sc into both flanks of four 8-week old NOD/SCID mice under anesthesia. After suture, mice were maintained under pathogen-free conditions as described before (first generation PDX). Tumor growth was monitored twice a week with an electronic caliper and after reaching a volume of about 600 mm^3^, tumors were collected by surgery under anesthesia, divided again into fragments of ~3 mm^3^ and implanted sc into the left flank of 10 8-weeks old NOD/SCID mice (second generation PDX).

### Cell culture

The urinary bladder cancer cell lines RT-4 (wild-type p53, mutant CDKN2A and TSC1), T24 transitional cell papilloma (p53- and HRAS-mutated) and transitional cell carcinoma UMUC-3 (p53-, CDKN2A-, KRAS- and PTEN-mutated) were purchased from ATCC (Manassas, VA, USA) and cultured according to seller´s guidelines. All cell cultures were maintained in DMEM containing 10% FBS (HyClone Laboratories, Logan, UT, USA) in a humidified cell culture chamber at 37°C and 5% CO_2_. Cultures were checked periodically for mycoplasma contamination using the EZ-PCR Mycoplasma Test Kit (Biological Industries Israel, Beit Haemek Ltd., Israel). All studies were performed within 2 years of cell purchase and cultures were discarded beyond 6 months after thawing.

To obtain primary cultures of normal human bladder epithelial cells (NBE), a fragment of normal tissue was removed after radical bladder surgery, transferred to the laboratory and dissected into 1-2 mm^3^ pieces. The tissue was washed three times in PBS at room temperature (RT) and digested for 4 h at 37ºC in RPMI containing 1 mg/ml collagenase I, 2 mg/ml collagenase IV, 1 mg/ml Dispase, 20 μg/ml hyaluronidase and 2000 U/ml DNase I. The cell suspension was centrifuged at 200 x *g* for 5 min at RT and the pellet was suspended in PBS and again centrifuged for 5 min at 200 x *g*. The final pellet was suspended in RPMI containing 5% FBS and seeded onto collagen I-coated T25 flasks (Nunc, Walthan, MA, USA) and cultured under normal conditions. Fibroblasts were removed by differential trypsinization^10^,^21^.

### Fluorescent *in situ* hybridization (FISH)

Fluorescence detection of SncmtRNA and AsncmtRNAs in cells was performed in suspension according to the protocol described before^22^.

### Chromogenic *in situ* hybridization

For tissue samples, 5-μm thick serial paraffin sections were collected onto silanized slides (DAKO, Sta Clara, CA, USA) and deparaffinized in 2 consecutive 5 min xylene washes. One section was stained with hematoxylin and eosin (H&E). The others were rehydrated in two 3-min washes of 98% and 90% ethanol each and once in DEPC-treated distilled water for 5 min^14^,^15^. Sections were then incubated in 2.5 μg/ml Proteinase K (Invitrogen, Carlsbad, CA, USA) at RT for 20 min and then washed twice for 3 min in DEPC-treated water, immersed in 96% ethanol for 10 s and air-dried. *In situ* hybridization was carried out as described before^16^ using 35 pmoles/ml digoxigenin-labeled probes targeted to SncmtRNA (5' TGATTATGCTACCTTTGCACGGT) and the ASncmtRNAs (5' ACCGTGCAAAGGTAGCATAATCA) in hybridization solution. Washing and color development were carried out as described before^14^,^15^.

### Cell transfection

Cells were seeded into 12-well plates (Nunc) at 50,000 cells/well and transfected the next day with 100 nM each ASO, using 2 µg/ml Lipofectamine2000 (Invitrogen). Transfection was allowed to proceed for 24-72 h under normal culture conditions. ASOs contained 100% phosphorothioate internucleosidic linkages (LGC Biosearch Technology Inc, Novato, CA. USA). The sequences of the ASOs used were 5' CACCCACCCAAGAACAGG (Andes-1537) and 5' TTATATTTGTGTAGGGCTA (non-related control ASO or ASO-C). To assess transfection efficiency, the same ASOs were labeled at the 5' end with Cy-3 (IDT Inc, Corallville. IA, USA).

### Reverse transcription polymerase chain reaction (RT-PCR)

Total RNA was extracted from cells with TRIzol reagent (Invitrogen), according to manufacturer´s instructions. cDNA synthesis was carried out with 100 ng RNA and MMLV reverse transcriptase as described before^15^,^16^. PCR reactions were prepared as described^14^,^15^ and amplification was performed as follows: 100°C for 10 min, 70°C for 10 min, 80°C for 10 min and 94°C for 5 min, followed by 26 cycles (S and ASncmtRNAs) or 16 cycles (18S rRNA), consisting of 94°C for 1 min, 58°C for 1 min and 72°C for 1 min. Primers used were 5' ACCGTCCAAAGGTAGCATAATCA (forw) and 5' CAAGAACAGGGTTTGTTAGG (rev) for ASncmtRNA-2; 5' TAGGGATAACAGCGCAATCCTATT (forw) and 5'CACACCCACCCAAGAACAGGGAGGA (rev) for ASncmtRNA-1; and 5'GTAACCCGTTGAACCCCATT (forw) and 5'CATCCAATCGGTAGTAGCG (rev) for 18S rRNA.

### Cell viability and proliferation assessment

Total cell number and viability was determined by Trypan blue (Tb) or propidium iodide (PI) exclusion. PI was added at 50 μg/ml 1 min before flow cytometry on a BD-FACS Canto Flow Cytometer (Fundación Ciencia & Vida). For Tb, the number of viable and dead cells was determined counting at least 100 cells per sample in triplicate under an Olympus BX-53 fluorescence microscope. Cell proliferation was assessed by using the 3-[4,5-dimethylthiazol-2-yl]-2, 5 diphenyl-tetrazolium bromide (MTT) (Promega, Fitchburg, WI, USA) assay. Briefly, cells were seeded in 96-well plates at 3,000 cells/well. At 0, 24, 48 and 72 h post-transfection, 20 µl/well of 5 mg/ml MTT was added and incubated for 2 h at 37°C. DMSO was then added (150 µl/well) and incubated for 10 min at RT. Absorbance was measured at 570 nm in an Epoch microplate reader (Biotek, Winossky, VT, USA).

### Mitochondrial depolarization (ΔΨm)

Cells were seeded in 12-well plates and transfected for 24 h as described above. Afterwards, cells were incubated with 20 nM tetramethylrhodamine methyl ester (TMRM; Molecular Probes, Eugene, OR, USA) for 15 min at 37°C, harvested and analyzed by flow cytometry on a BD-FACS canto Flow Cytometer^8^,^10^. As positive control, mitochondrial depolarization was elicited using 10 µM carbonyl cyanide 3-chlorophenylhydrazone (CCCP; Sigma Aldrich, St. Louis, MO, USA) for 30 min at 37°C.

### Western blotting

Proteins were extracted from cells as described^8^ and protein concentration was determined via standard bicinchoninic acid assay (BCA) protocol (Pierce, Rockford, Rockford, IL. USA). Cell lysates (30 µg) were subjected to SDS-PAGE and transferred to polivinylidene difluoride (PVDF) membranes on a Transblot- Turbo Transfer System (Bio-Rad, Hercules, CA, USA) at 25 V for 17 min. Membranes were incubated overnight at 4ºC in blocking solution [5% non-fat dry milk, 0,1% Tween 20 in 1X Tris-buffered saline (TBS)]. On the following day, membranes were incubated with rabbit polyclonal anti E-cadherin (1:1000; BD Biosciences, Franklin Lakes, NJ, USA), N-cadherin (1:1500; BD Biosciences), MMP-11 (1:2000; R&D systems, Minneapolis, MN, USA), procaspase-3 (1:1000; Abcam, Cambridge, UK), Bcl-xL (1:500; Abcam) or survivin (1:5000; Abcam), in blocking solution overnight at 4°C. After 3 washes for 5 min at RT in TBST (0,1% Tween-20 in TBS), and membranes were incubated with HRP-conjugated anti-rabbit IgG (1:2000; KPL, Milford, MA, USA) for 1h at RT. For loading controls, membranes were probed with mouse monoclonal anti-β-actin (1:4000; Sigma-Aldrich) or anti-GAPDH (1:5000; R&D Systems), followed by HRP-conjugated anti-mouse IgG (1:2000; KPL) for 1h at RT. After 3 washes in TBST, blots were revealed with the EZ-ECL system (Biological Industries) on a C-DiGit blot Scanner (LI-COR Biosciences, Lincoln, NE, USA). The pixel intensity of each protein band was quantified using Image J software (NIH).

### Determination of Phosphatidylserine (PS) exposure

PS exposure was assessed by Annexin-V binding with the AlexaFluor®488-Annexin V/dead Cell apoptosis Kit (Molecular probes), according to manufacturer's instructions and analyzed by flow cytometry^8^-^11^.

### Terminal deoxynucleotidyl transferase dUTP nick end labeling (TUNEL) assay

Apoptosis was determined by TUNEL, using the Fluorometric *in situ* Cell Death Detection kit (Promega), according to manufacturer's instructions. Ten high-magnification fields were counted and the proportion of apoptotic (green) cells was determined, as the fraction of total cells. For assessment of apoptosis *in vivo* after Andes-1537 treatment, tumors from ASO-C- or Andes 1537-treated mice were surgical resected, fixed immediately in neutral-buffered formalin (10%) and paraffin-embedded. Afterward, 5 μm-thick serial paraffin sections were collected on silanized slides (DAKO) and hydrated as described before^11^,^15^. The TUNEL procedure was performed using DeadEnd™ Colorimetric Apoptosis Detection System (Promega), according to manufacturer's instruction. As positive control, DNAse I-treated muscle and liver sections were included. Control tumor sections were counter-stained with contrast blue (KPL).

### Stemness and invasion of UMUC-3 cells

To determine stemness^23^,^24^, 5,000 Tb-negative UMUC-3 cells transfected as described above for 24 h were suspended in MEGM (Lonza) supplemented with 25 ng/ml EGF, 5 mg/ml hydrocortisone, 5 µg/ ml insulin (Lonza) and 25 ng/ml bFGF (Invitrogen) and seeded into 2% agarose-coated 12-well-plates. After incubation at 37°C for 12 days, spheres >50 μm in diameter were scored. For matrigel invasion assay, 10^5^ cells treated as above for 48 h were seeded over Matrigel-coated inserts (Matrigel Invasion Chamber 8.0 lm; BD Biosciences). After 24 h, inserts were fixed in 4% formaldehyde, and membranes were stained with DAPI, mounted in Mowiol and observed under an Olympus CKX41 microscope at 40X magnification. At least 10 fields were evaluated^10^.

### Statistical Analysis

Experimental results were analyzed by Student's *t*-test and significance (*p*-value) was set at the nominal level of p<0.05 or less.

## Results

### Differential expression of the SncmtRNA and ASncmtRNAs in BCa and normal bladder cells

**Figure 1:**
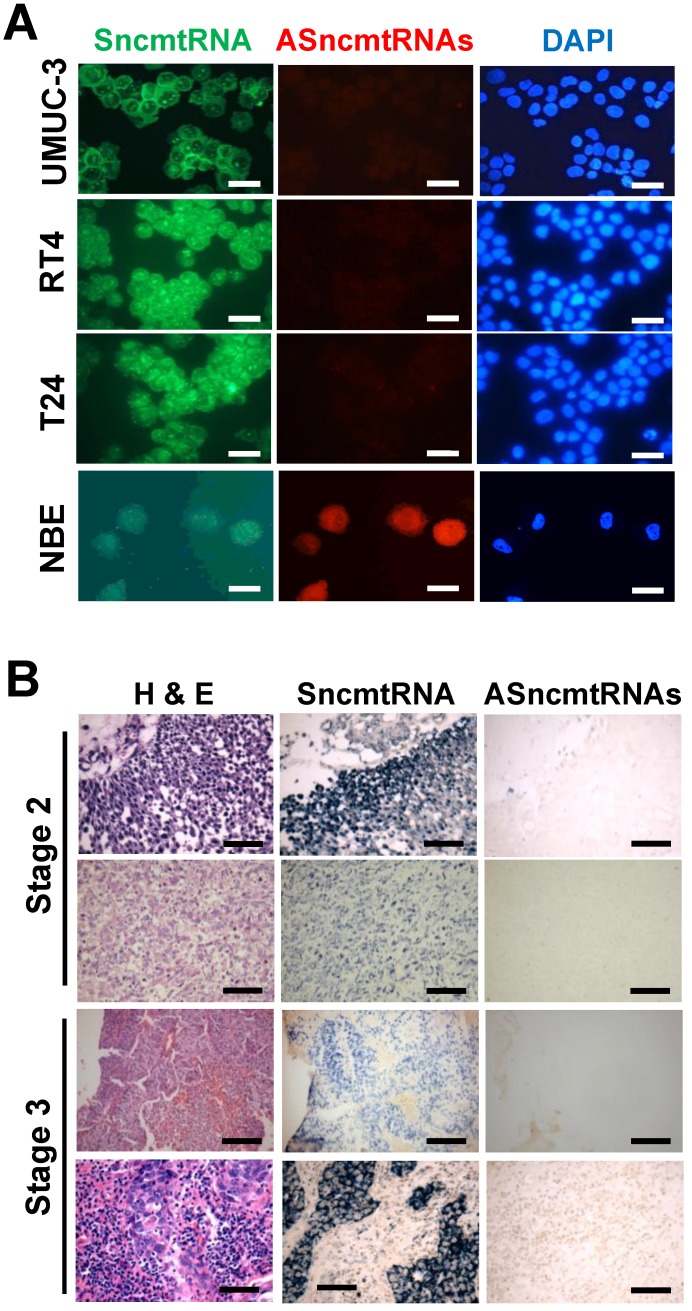
** Differential expression of ncmtRNAs in bladder cancer cell lines and biopsies**. (A) FISH of UMUC-3, RT-4 and T24 cells shows expression of SncmtRNA (green) and downregulation of ASncmtRNAs (red). NBE cells express both mitochondrial transcripts. Nuclei were counterstained with DAPI (blue) (Bars = 25 µm). (B) Four sections of each BCa biopsy (Grade 2 and 3) were stained with Hematoxylin/Eosin (H&E) and subjected to chromogenic ISH using specific probes for SncmtRNA and ASncmtRNAs, respectively (Bars = 100 µm).

As observed previously for other human tumor cell lines, FISH of UMUC-3, RT4 and T24 human BCa cell lines revealed expression of the SncmtRNA and downregulation of the ASncmtRNAs (Fig. 1A). In contrast, normal epithelial cells obtained from healthy bladder tissue (NBE), express both mitochondrial transcripts (Fig. 1A). Chromogenic *in situ* hybridization of biopsies showed that the ASncmtRNAs are also downregulated in BCa tumors, independent of the malignancy grade (Fig. 1B).

### ASK induces strong inhibition of proliferation of bladder cancer cells

**Figure 2:**
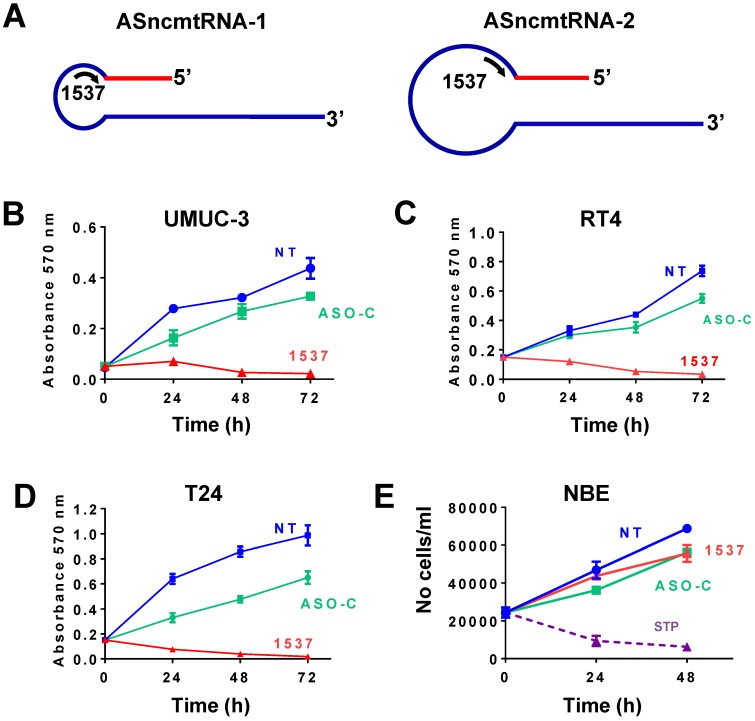
** ASK induces strong inhibition in proliferation of BCa tumor cell lines**. (A) Schematic representation of ASncmtRNA-1 and -2, indicating the complementary position of Andes-1537 (1537S). (B-D) UMUC-3 (B), RT4 (C) and T24 cells were transfected with Andes 1537 or ASO-C or left untreated for 24, 48 and 72 h and cell viability was determined at each time point by MTT assay. (E) Normal bladder epithelial (NBE) cells were transfected as described before for 24 and 48 h and cells were stained with Trypan blue. As positive control, NBE cells were incubated with 1 uM staurosporin (STP).

UMUC-3, RT4 and T24 cells were transfected with Andes-1537, targeted to the loop of the ASncmtRNAs (Fig. 2A). Similarly, to other human and mouse tumor cell lines^12^, ASncmtRNA knockdown (ASK) elicited in this manner induced a drastic inhibition of proliferation in all 3 BCa cell lines, compared to controls (Fig. 2B-D). In contrast, proliferation of normal NBE cells was not affected by the same treatment, compared to the strong reduction in viability induced by staurosporin (STP)^26^ (Fig. 2E).

### ASK induces apoptosis of bladder cancer cells

**Figure 3:**
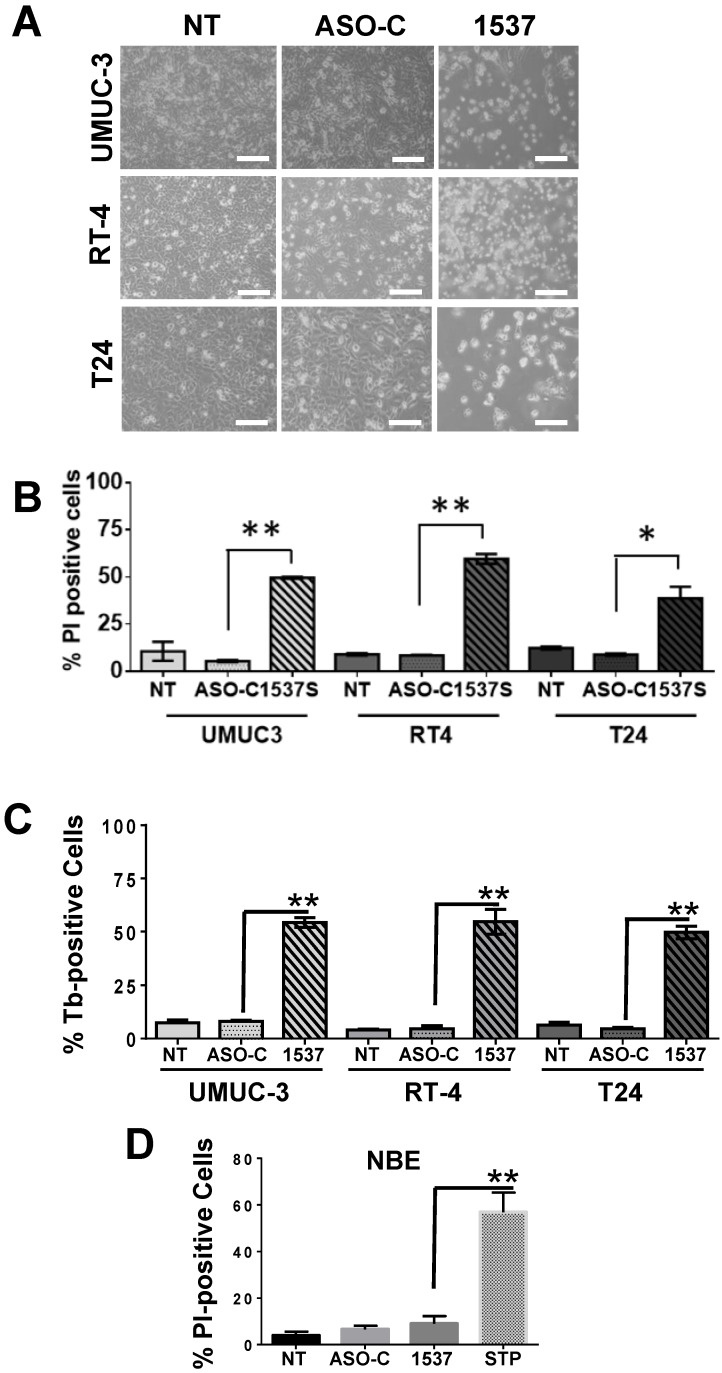
** ASK induces cell death of BCa cell lines**. UMUC-3, RT4 and T24 cells were transfected for 48 h with Andes-1537 or ASO-C or left untreated (NT). (A) Andes-1537 caused massive cell detachment from the substrate, as opposed to controls (NT, ASO-C) (Bars = 50 μm). (B, C) Andes-1537 induced 50-60% death in all 3 cell lines, compared to controls, as evidenced by Tb (B) and PI (C) exclusion. *p<0.05, **p<0.01, Andes-1537 vs. ASO-C. (D) Normal bladder epithelial cells were transfected with Andes-1537 or ASO-C or left untreated for 48 h and permeability to PI was determined by cytometry. Andes-1537 treatment displayed no significant level of cell death, like controls. Staurosporine (STP) treatment was used as positive control. **p<0.01, STP vs. Andes-1537.

UMUC-3, RT4 and T24 cells transfected with Andes-1537 exhibited massive detachment from the substrate at 48 h, as compared to cells transfected with ASO-C or left untreated (NT) (Fig. 3A). This detachment and loss of cell viability (Fig. 3B-C) were the result of increased cell death, since ASK induced 50-60% Trypan blue (Tb)-positive (Fig. 3B) and PI-positive (Fig. 3C) cells. In contrast, normal bladder epithelium (NBE) cells treated with Andes-1537 did not display a higher proportion of PI-positive cells compared to controls (Fig. 3D), although, as expected, staurosporin induced around 70% PI-positive cells (Fig. 3D).

**Figure 4:**
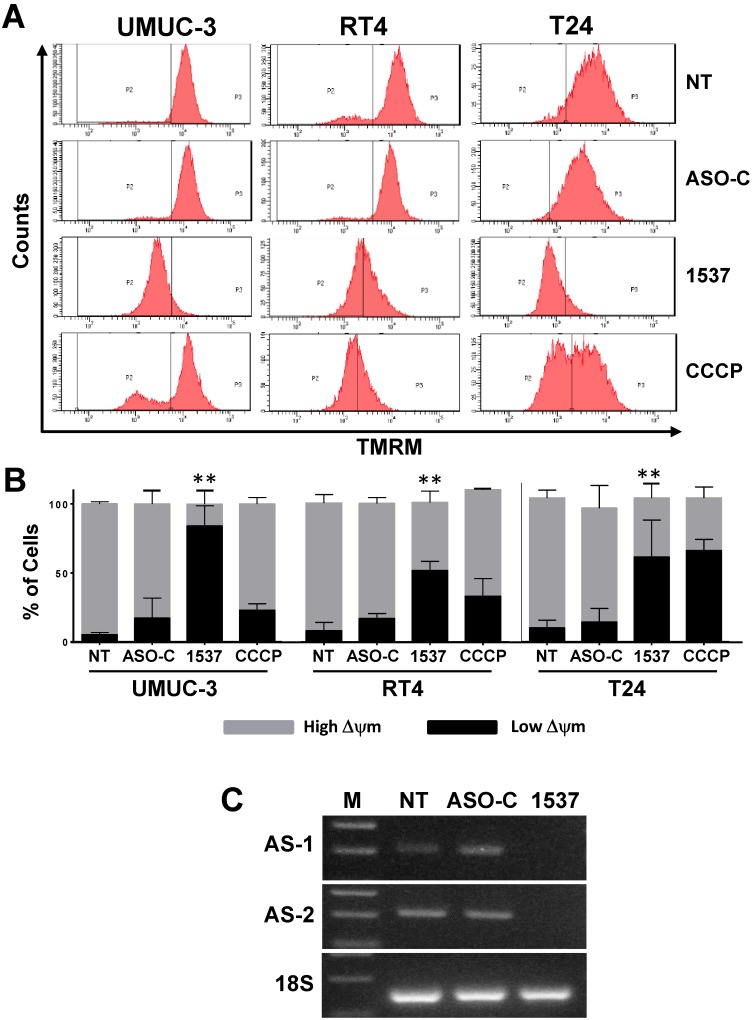
** ASK induces dissipation of mitochondrial membrane potential (Δψm).** UMUC-3, RT4 and T24 cells treated with Andes-1537 or ASO-C, or left untreated (NT) for 24h or with uncoupling drug CCCP were harvested at 24 h post-transfection, stained with 20 nM TMRM for 15 min and analyzed by flow cytometry, revealing that ASK induces dissipation of Δψm. (B) A triplicate analysis shows that Andes-1537 induced between 50 and 80% dissipation of Δψm (UMUC-3, **p=0.0073; RT4, **p=0.0024; T24, p=0.017). (C) RT-PCR of total RNA purified from cells treated as in (A) for 48 h revealed the specific knockdown effect of Andes-1537 on ASncmtRNA-1 (AS-1) and ASncmtRNA-2 (AS-2). 18S rRNA was used as internal control. (M, 100-bp ladder).

In order to determine if ASK-induced death of BCa cell lines occurs through apoptosis, as observed previously for other cancer cell types^11^, we measured mitochondrial membrane potential (ΨΔm)^26^-^28^ in UMUC-3, RT4 and T24 cell lines treated as above for 48 h and stained with tetramethyl-rhodamine methylester (TMRM). The uncoupling drug CCCP was used as positive control^26^. Flow cytometry revealed that ASK induced a marked dissipation of ΨΔm in all 3 cell lines, compared to controls (Fig. 4A). Results of three independent experiments show that Andes-1537 induced 50-80% dissipation of ΨΔm (Fig. 4B). Note that CCCP induces only 40% ΨΔm dissipation. RT-PCR corroborated that ASK induced knockdown of ASncmtRNA-1 and ASncmtRNA-2 targets (Fig. 4C).

**Figure 5:**
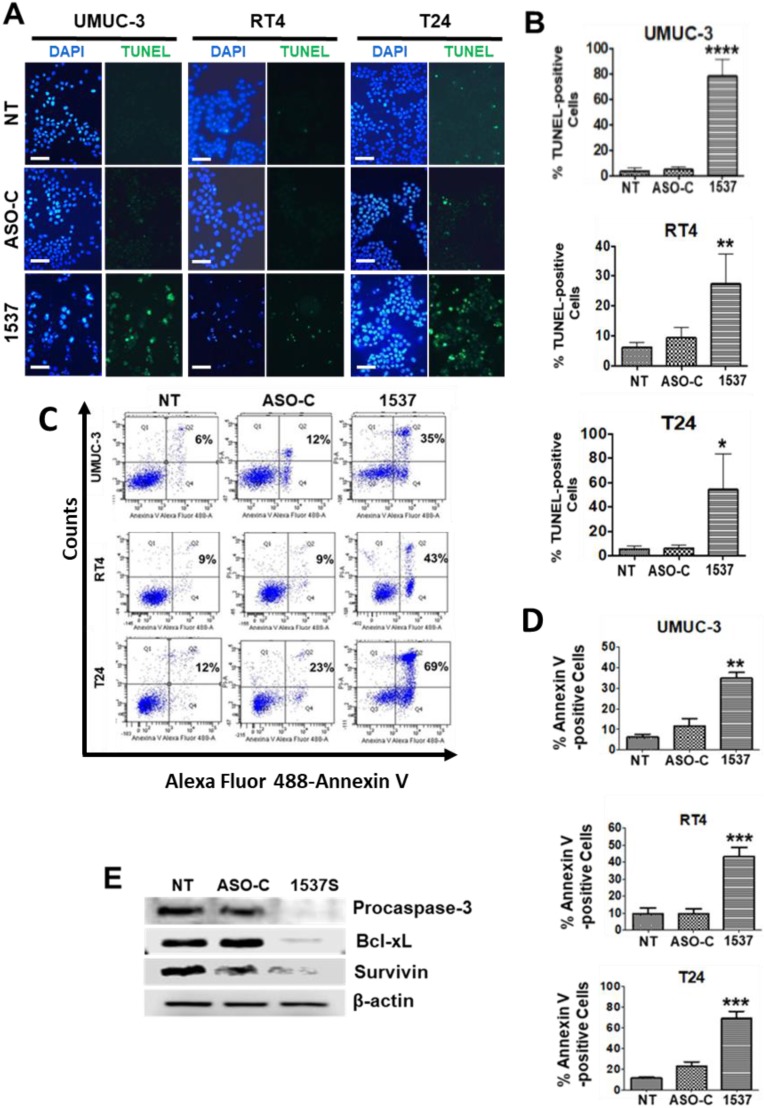
** ASK induces chromatin fragmentation and phosphatidylserine translocation in BCa cell lines**. (A) UMUC-3, RT4 and T24 cells were transfected with Andes-1537 or ASO-C or left untreated (NT) for 48 h and subjected to TUNEL assay. Andes-1537 transfection induced an increase in TUNEL-positive cells. Bars = 50 µm. (B) Quantification of TUNEL positive cells in in all 3 lines. (C) BCa cell lines were analyzed by flow cytometry, revealing a higher population of Annexin V-positive cells in samples treated with Andes-1537. (D) Quantification of Annexin V-positive cells in the three cell lines, as compared to controls (NT and ASO-C). (E) Total lysates of UMUC-3 cells treated as in (A) were subjected to Western blot, revealing triggering of apoptosis through cleavage of procaspase-3 and drastic downregulation of Bcl-xL and survivin by ASK, compared to controls (NT and ASO-C). *p<0.05; **p<0.01; ***p<0.001; ****p<0.0001; Andes-1537 vs. ASO-C.

In addition, ASK for 48 h induced a strong increase in chromatin fragmentation (Fig. 5A, B), as evidenced by TUNEL assay, and translocation of phosphatidylserine to the outer layer of the plasma membrane (Fig. 5C, D), both hallmark markers of apoptosis^25^.

### ASK induces downregulation of the anti-apoptotic factors survivin and Bcl-xL

Apoptotic cell death depends on the counterbalance between pro-and anti-apoptotic factors^29^,^30^. Western blot of UMUC-3 cells transfected as described above for 48 h showed that ASK induces cleavage of procaspase-3 (Fig. 5E), in accordance with the apoptotic effects described above. ASK also induces downregulation of the anti-apoptotic factors Bcl-xL^31^, and survivin (Fig. 5F), a member of the inhibitor of family of inhibitors of apoptosis (IAP) proteins^12^.

### ASK reduces stemness and invasiveness of BCa cell lines

**Figure 6:**
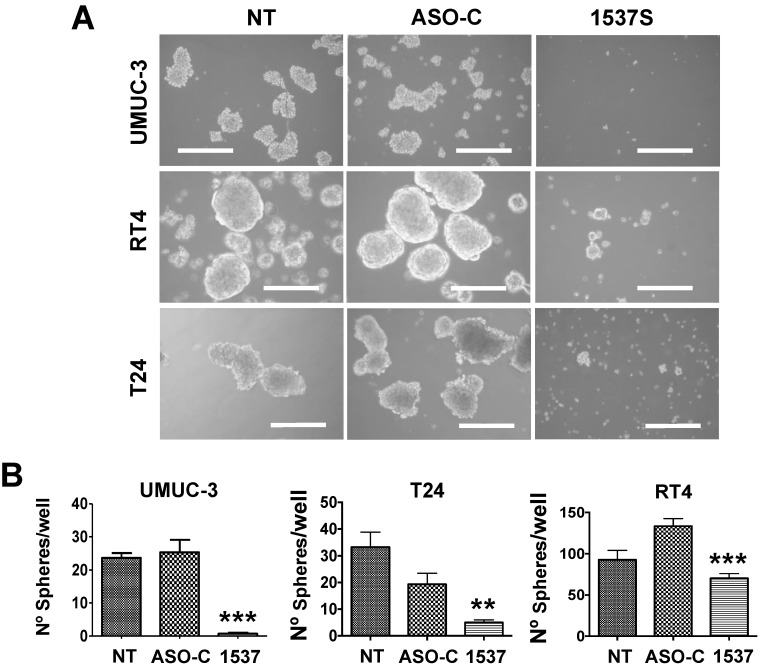
** ASK induces inhibition of stemness in BCa cell lines**. UMUC-3, RT4 and T24 cells (50,000 cells/well) were transfected with Andes-1537 or ASO-C or left untreated (NT) for 48 h. After harvesting, 5,000 Tb-negative cells were seeded into 12-well agarose-coated plates and cultured for 10-12 days, when spheres >50 µm in diameter were scored. (A) Representative phase contrast images of spheres (Bars = 200 µm). (B) A triplicate analysis showed that ASK induces between 50 and 90% inhibition of sphere formation in cell lines. **p<0.01, ***p<0.001.

An important property of tumorigenicity is the presence of cancer stem cells (CSC), a small sub-population of tumor cells that are responsible for the initiation, progression, local and distant metastasis, resistance to chemo- and radiotherapy and disease relapse^32^-^34^. Therefore, anchorage-independent sphere formation is an important *in vitro* parameter to determine stemness of tumor cells. To determine whether ASK affects stemness, UMUC-3, RT4 and T24 cells were transfected with Andes-1537 or ASO-C or left untreated (NT) for 48 h. Cells were then harvested, counted and 5,000 Tb-negative cells were seeded per well into agarose-coated 12-well plates (see Methods). After 10 days in culture, spheres >50 µm in diameter were scored, revealing that ASK strongly inhibits sphere formation (Fig 6A, B).

**Figure 7:**
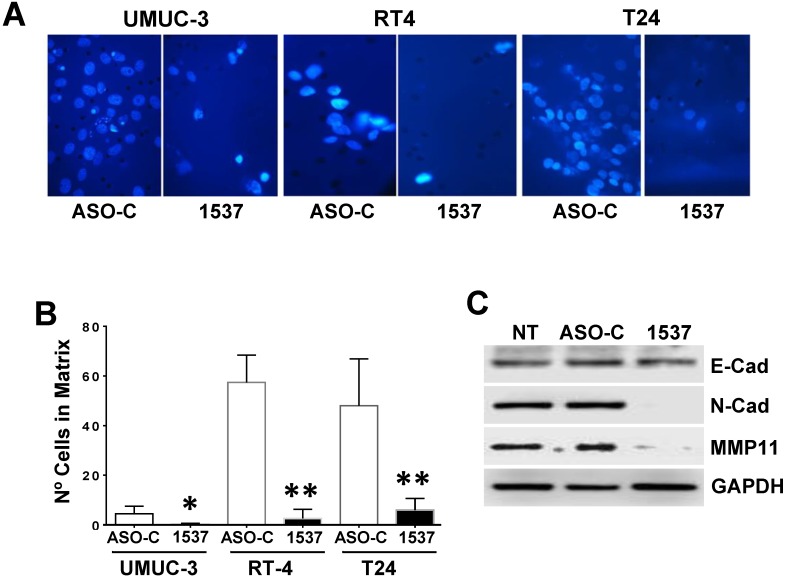
** ASK reduces invasiveness of BCa cell lines**. UMUC-3, RT4 and T24 cells (50,000 cells/well) were transfected with Andes-1537 or ASO-C or left untreated (NT) for 48 h. (A) Invasion was determined with Matrigel Transwell Assay. (B) A triplicate analysis showed that ASK induces a drastic and significant reduction of invasiveness in all 3 cell lines. *p<0.05, **p<0.01. (C) UMUC-3 cells transfected as in (A) for 48 h were collected and total lysate was subjected to Western blot analysis of E-cadherin (E-cad), N-cadherin (N-cad) and Matrix Metallopeptidase 11 (MMP), using GAPDH as loading control. ASK induces down regulation of N-cadherin and MMP11.

To determine whether ASK affects the invasive capacity of UMUC-3 cells^8^, after a 24 h treatment as described above, 150,000 Tb-negative cells were seeded over Matrigel-coated inserts. After 24 h, the membranes were fixed, mounted in Mowiol, stained with DAPI and observed by fluorescence microscopy. ASK induced a significant reduction on the invasion capacity of UMUC-3 cells (Fig. 7A, B). Western blot showed that this effect is brought about by downregulation of N-cadherin and Matrix Metallopeptidase 11 (MP11), key proteins involved in epithelial-mesenchymal transition (EMT)^32^-^34^.

### ASK inhibits growth of UMUC-3 tumors *in vivo*

**Figure 8:**
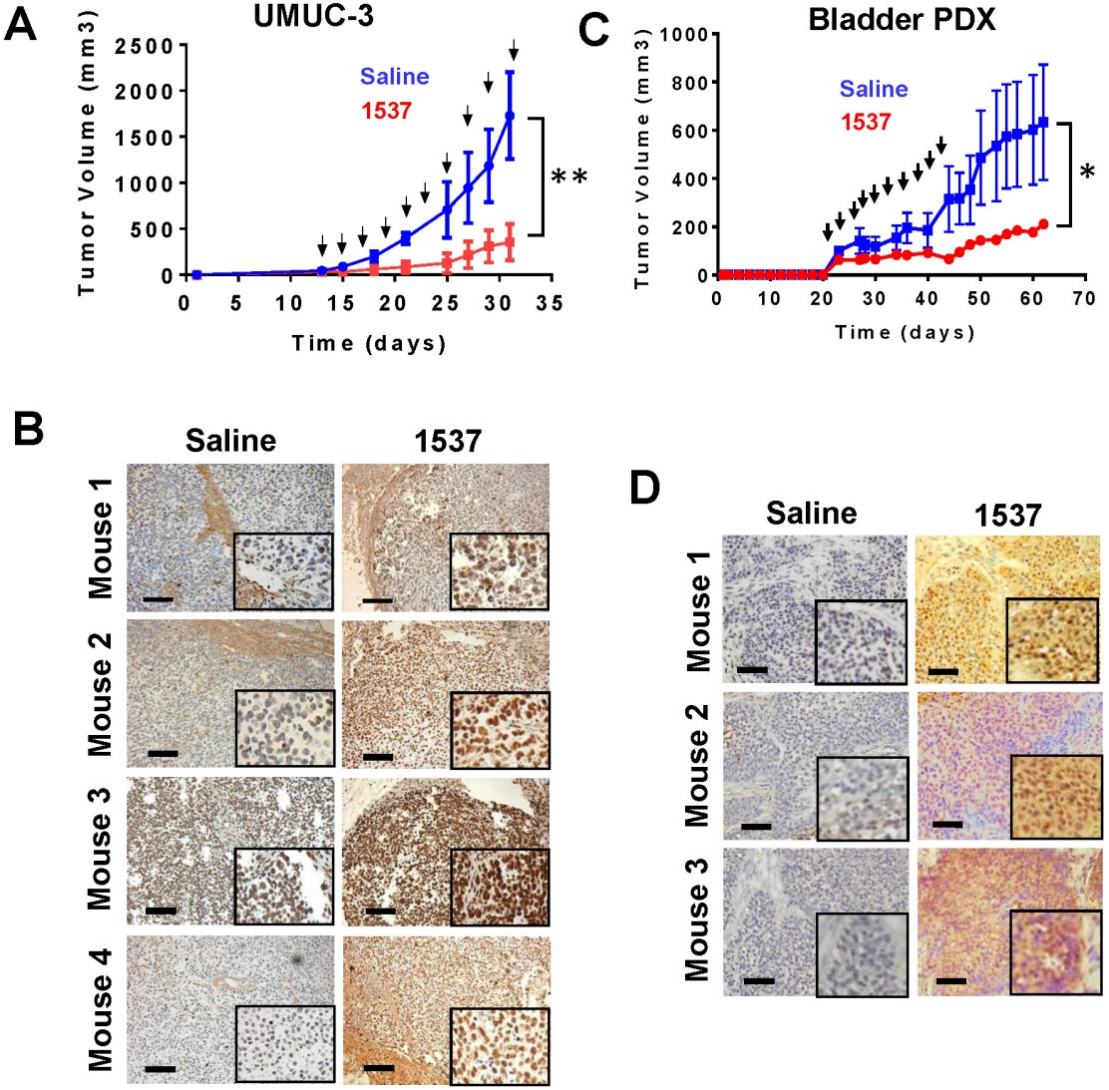
** ASK delays BCa tumor growth *in vivo***. (A) Mice bearing tumors between 50-80 mm^3^ were randomized into 2 groups of 5 mice each, which were treated with Andes-1537 or saline alone. At 32 days post-cell injection, ASK induced a drastic inhibition of tumor growth (**p=0.0031), mice were euthanized under anesthesia and tumors were collected from both groups. (B) ASK induces TUNEL-positive cells in tumors. Only tumors from Andes-1537-treated mice showed positive TUNEL nuclear staining (Bars = 250 µm). Inserts show higher magnification of the same samples. (C) Andes-1537S inhibits growth of a BCa PDX tumor. On day 23 post-implantation, tumors of about 100 mm3 were observed and mice were randomized into two groups. The control group was injected ip with 200 µl saline and the treatment group with 100 µg Andes-1537. The remaining 9 injections of the treatment group were performed with 50 µg Andes-1537 (arrows). ASK induced significant inhibition of tumor growth, compared to saline (*p=0.0078). (D) At day 62 post-implantation, mice were euthanized under anesthesia and tumors sections were analyzed by TUNEL assay, showing that tumors from Andes-1537-treated PDX mice displayed positive nuclear signal (Bars = 250 µm).

In order to probe *in vivo* the effect of ASK on BCa tumor growth, we injected UMUC-3 cells (5 x 10^5^) sc into the left flank of 10 NOD-SCID mice. When tumors reached an average volume about 80 mm^3^, mice were randomized into two groups of 5 mice each, with similar tumor size. Mice were injected intraperitoneally (ip) every other day, in a blinded fashion, with 10 doses of 200 µl saline either alone or containing 100 µg Andes-1537 (see arrows Fig. 8A). Tumor growth of saline-injected mice reached around 800 mm^3^ on day 30 post-cell injection, while tumor growth in mice treated with Andes-1537 was strongly inhibited, reaching a maximum average tumor size of 180 mm^3^ (Fig. 8A). On day 32 post-cell injection, all mice were sacrificed under anesthesia and tumors were collected, fixed and cut into 5 µm sections. To assess the effect of treatment on induction of apoptosis, tumor sections were analyzed by TUNEL assay. Around 70% of TUNEL-positive nuclei was observed in tumor sections from mice treated with Andes-1537, in contrast with absence of signal in saline control tumors (Fig. 8B, see inserts).

### Evaluation of ASO therapy in a patient derived-xenograft (PDX) model of bladder cancer

To evaluate the effect of ASK *in vivo* in a scenario closer to real clinical specimens, we developed a PDX BCa model. The tumor sample was obtained from a male BCa patient at stage T2N0M0^35^, displaying penetration through the muscle layer. Small pieces of the tumor sample of about 3 mm^3^ were implanted sc in 3 mice and after 3 weeks, tumors ~600 mm^3^ were collected after surgery under anesthesia (first generation PDX). This first generation of PDX was again divided in pieces of about 3 mm^3^ and implanted sc into 10 mice (second generation PDX). When tumors reached a size between 80 and 100 mm^3^, mice were randomized into two group of 5 mice each and injected ip with 200 μl saline, either alone or containing 100 μg Andes-1537. This first injection was followed by 9 injections with saline alone or containing 50 μg Andes-1537 (see arrows Fig. 8B). Treatment with Andes-1537 induced strong inhibition of tumor growth as compared to the control group (Fig. 8C). On day 62 post-tumor implantation, mice were sacrificed under anesthesia and tumors of three mice were collected, fixed and sectioned. Chromogenic TUNEL assay revealed around 60% positive nuclei in tumors from Andes-1537-treated mice, compared to no signal in the control group (Fig. 8D).

## Discussion

As described previously for other cancer cell types^11^, the mitochondrial ASncmtRNAs, as well as SncmtRNA, are expressed in normal proliferating bladder cells, but the former is downregulated in BCa cell lines as well as in BCa biopsies^15^. Interestingly, we reported that the urine from patients with BCa contain cells that show expression of SncmtRNA and downregulation of ASncmtRNAs, in contrast to urine from healthy donors^22^. Hence, downregulation of the ASncmtRNAs seems to be an important step during carcinogenesis and represents a general hallmark of cancer^11^,^15^. Interestingly, in mouse cells the expression pattern of these mitochondrial transcripts is identical to human cells and mouse tumor cells also downregulate the ASncmtRNAs^8^,^10^. The mechanism underlying the downregulation of these transcripts during carcinogenesis is unclear, but in human papilloma-virus infected cells (HPV), it appears to be mediated by the E2 viral oncogene^16^. Interestingly, mouse hepatocytes transfected with a vector encoding hepatitis B virus antigen X also display downregulation of the ASncmtRNAs (E. Jeldes, unpublished results). However, the important question of which factor(s) induce(s) downregulation of these mitochondrial transcripts in non-viral induced cancers remains unknown.

As reported here, knockdown of the ASncmtRNAs (ASK) with Andes-1537 induces strong inhibition of cell proliferation and apoptotic cell death of UMUC-3, RT4 and T24 BCa cell lines, independent of their genetic backgrounds (see Methods). In UMUC-3 cells, apoptosis is mediated at the molecular level by downregulation of Bcl-xL and survivin, two important apoptotic inhibitors^25^,^31^. In other words, BCa cells cannot elude apoptotic death and, more importantly, the ASO treatments do not affect viability of normal bladder epithelial cells. Probably the lower copy number of ASncmtRNA in tumor cells renders them more vulnerable to ASK, although this hypothesis warrants further research^15^. Therefore, a question that remains unanswered is why tumor cells maintain low levels of ASncmtRNAs. Does downregulation of ASncmtRNAs constitute a kind of oncogenic switch? We believe that the high levels of these RNAs in normal cells could play a tumor suppressor role. This dual role of a molecule in carcinogenesis and metastasis has been described for mutated forms of p53^36^,^37^, and TGF-β during breast cancer progression^38^-^39^.

In addition to apoptotic cell death, ASK also induces strong inhibition of stemness and invasion^40^,^41^ (Fig. 6, 7). Inhibition of invasiveness correlates with downregulation of N-cadherin and MMP11, two important factors involved in EMT, invasion and metastasis^43^. An important question is how Andes-1537 can reduce the levels of these 2 EMT-promoting factors and 2 apoptosis inhibitors (survivin and Bcl-xL). We hypothesized that in human melanoma SK-MEL-2 cells, downregulation of similar proteins by ASK might be mediated by microRNAs (miRNAs)^11^, which have become increasingly important amongst epigenetic factors controlling tumorigenesis^43^,^44^. We postulated that these miRNAs are probably generated by Dicer-mediated processing of the double-stranded stem of the ASncmtRNAs as a consequence of ASK^11^. Recently, we found that ASK induces the expression of hsa-miR-1973 and hsa-miR-4485-3p in the breast cancer cell line MDA-MB-231, with sequences that match perfectly to the sequence of the stem of ASncmtRNA-2. In addition, nuclear-encoded miRNAs are also upregulated by the treatment^45^.

Previously we showed that ASK is an effective *in vivo* strategy to reduce tumor growth in murine syngeneic models of melanoma (B16F10 cells)^8^,^9^ and renal cell carcinoma (RenCa cells)^10^. Patient-derived xenograft (PDX) tumors are models developed by direct transplantation of human tumor fragments into immune-compromised mice. In BCa, recently a total of 65 human bladder cancer tissues samples were implanted sc into the flanks of immunodeficient mice, obtaining six successful PDX models, and primary tumor tissues and xenografts of passage 2 or higher were compared. The analysis showed similar histological characteristics, degree of differentiation, and genomic alterations between the primary and xenograft tumors^46^. Moreover, previous work reported that PDX models allowed efficacy studies of combination therapy, either concurrent or sequential, to select the most effective therapy to be used in patients^47^. For these reasons, PDX models are considered a valuable platform for translational cancer research and are becoming standard practice for cancer research^48^-^50^.

*In vivo* results in xenografts and in the PDX model, support the proof-of-concept of the potential use of ASK for BCa therapy. Indeed, a Phase I Clinical Trial (NCT02508441) with Andes-1537 in San Francisco, CA (UCSF) was recently completed. Andes-1537 was well-tolerated and one patient with pancreatic cancer and another with cholangiocarcinoma maintained stable disease beyond six months^51^.

## Conclusions

Our study shows that knockdown of ASncmtRNAs induces a pleiotropic effect, affecting simultaneously several physiological processes such as proliferation, apoptosis evasion and invasion, required for bladder cancer progression. However, there are still some limitations in our study, for example, whether ASK is responsible for the generation of miRNAs that act negatively on several different targets. Or, can the ASncmtRNAs act as miRNA sponges or ceRNAs? Precise molecular mechanisms underlying the effects of ASK by antisense treatment and the negative effects over bladder tumor cell physiology warrants future research.

## Abbreviations

ASncmtRNAs: antisense non-coding mitochondrial RNAs
ASK: ASncmtRNA knockdown
ASO: antisense oligonucleotide
BCa: bladder cancer
SncmtRNA: sense non-coding mitocondrial RNA
PDX: patient-derived xenograft
